# Anti‐Inflammatory Effects of Fermented and Aged Mountain‐Cultivated Ginseng Sprouts via Suppression of MAPK‐NF‐κB Pathway in Lipopolysaccharide‐Stimulated RAW264.7 Macrophages

**DOI:** 10.1002/fsn3.4518

**Published:** 2024-10-18

**Authors:** Dang Long Cao, Min‐Seok Woo, Eun‐Jin Kim, Byeonggyu Ahn, Anjas Happy Prayoga, Sang Soo Kang, Kye Man Cho, Dawon Kang

**Affiliations:** ^1^ Department of Physiology College of Medicine, Gyeongsang National University Jinju Republic of Korea; ^2^ Department of Convergence Medical Science Gyeongsang National University Jinju Republic of Korea; ^3^ Institute of Medical Sciences Gyeongsang National University Jinju Republic of Korea; ^4^ Department of Anatomy, College of Medicine Gyeongsang National University Jinju Republic of Korea; ^5^ Department of GreenBio Science and Agri‐Food Bio Convergence Institute Gyeongsang National University Jinju Republic of Korea

**Keywords:** fermented and aged mountain‐cultivated ginseng sprout, inflammation, macrophage, MAPK, NF‐κB

## Abstract

Fermented and aged mountain‐cultivated ginseng sprouts (FAMCGS) exhibit superior antioxidant and anti‐inflammatory properties compared to mountain‐cultivated ginseng sprouts (MCGS). However, the mechanisms behind these properties of FAMCGSE remain unclear. This study explores the anti‐inflammatory effects of FAMCGS extract (FAMCGSE) on LPS‐stimulated RAW 264.7 macrophages and the underlying mechanisms. The MTT assay confirmed that FAMCGSE (0 to 0.1%) maintained cell viability without inducing morphological changes. Pretreatment with FAMCGSE significantly mitigated LPS‐induced morphological alterations dose‐dependently. RT‐PCR and Western blot analyses showed that FAMCGSE significantly reduced the mRNA and protein levels of proinflammatory mediators such as TNF‐α, IL‐1β, IL‐6, iNOS, and COX‐2. Additionally, FAMCGSE decreased the production of TNF‐α, IL‐1β, IL‐6, nitric oxide, and PGE_2_ in LPS‐activated RAW264.7 cells. Mechanistically, FAMCGSE inhibited the phosphorylation of mitogen‐activated protein kinases (MAPKs; ERK, p38, and JNK) and prevented the LPS‐induced nuclear translocation of NF‐κB, with effects comparable to compound K (CK) or dexamethasone. Notably, FAMCGSE was particularly effective in inhibiting ERK and JNK activation, with less impact on p38, suggesting a specific inhibitory action on certain MAPK pathways. These findings highlight FAMCGSE's potential as an inhibitor of MAPK and NF‐κB pathways, indicating that FAMCGSE, including its main component CK, may be a promising therapeutic agent for inflammation‐related conditions.

## Graphical abstract

A schematic diagram illustrating the anti‐inflammatory mechanism of FAMCGSE in RAW264.7 cells exposed to LPS. The diagram shows how bacterial lipopolysaccharide (LPS) binds to Toll‐like receptor 4 (TLR4), triggering the activation of downstream MAPK and NF‐κB signaling pathways. These pathways lead to the transcription of proinflammatory mediators such as TNF‐α, IL‐1β, IL‐6, iNOS, and COX‐2, which drive inflammatory responses. FAMCGSE inhibits the activation of these pathways, thereby reducing the production of proinflammatory mediators and mitigating inflammation.

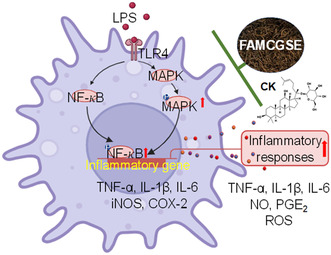

## 1 Introduction

Inflammation is an essential physiological response orchestrated by the immune system to protect the body from infections and injuries. This response is characterized by classic signs such as redness, swelling, heat, and pain, indicating the body's attempt to eliminate harmful stimuli and initiate the healing process (Chen et al. 2018; Hannoodee and Nasuruddin 2024). While acute inflammation is a protective mechanism, chronic inflammation can become detrimental when sustained over long periods. This prolonged inflammatory state is associated with various diseases, including cancer, cardiovascular disease, diabetes mellitus, chronic kidney disease, and non‐alcoholic fatty liver disease (Furman et al. 2019). Given these associations, controlling inflammatory responses is a significant area of interest in therapeutic research (Chen et al. 2018; Furman et al. 2019).

Macrophages play a central role in the inflammatory response by recognizing pathogens and releasing cytokines and chemokines. These signaling molecules, such as tumor necrosis factor‐alpha (TNF‐α) and interleukins (IL‐1β, IL‐6), are critical in mediating and regulating the inflammatory process, facilitating the recruitment and activation of additional immune cells at the site of injury (Fujiwara and Kobayashi 2005a; Hamidzadeh et al. 2017). When toll‐like receptor 4 (TLR4) is activated by lipopolysaccharide (LPS), a bacterial endotoxin, it triggers the activation of the mitogen‐activated protein kinase (MAPK) pathway and nuclear factor kappa B (NF‐𝜅B), leading to the production of inflammatory mediators (Chen et al. 2018; Li et al. 2022).

Targeting macrophage activation and cytokine production has been identified as a potential strategy for modulating inflammation, highlighting the need for further research into natural products and compounds that can effectively regulate these processes (Hamidzadeh et al. 2017). Among such natural products, ginsenosides in ginseng (*Panax ginseng*) have shown significant promise. Ginseng has long been a popular dietary supplement due to its various ginsenosides with pharmacological effects, including anti‐diabetic, anti‐inflammatory, antioxidant, lipid‐lowering, anti‐aging, and anti‐cancer properties (Li et al. 2023; Choi et al. 2013). This is especially true for wild ginseng (Ratan et al. 2021). Wild‐cultivated ginseng, which mimics the natural environmental stresses of forests, has excellent medicinal properties but requires a more extended cultivation period compared to cultivated ginseng (Liu et al. 2008; Park et al. 2019). Efforts are being made to develop processing methods using ginseng sprouts to shorten the cultivation period while maintaining the medicinal properties. In addition, sprouts contain more vitamins, enzymes, and nutrients necessary for growth than mature plants (Howell 1995). Effective bioactive substances are derived through fermentation and aging processes (Lee, Kim, and Kim 2015; Lee et al. 2021).

Fermented and aged mountain‐cultivated ginseng sprouts (FAMCGS) have shown notable increases in bioactive components, particularly the ginsenoside compounds K (CK), F2, and Rg3, through the processes of aging and fermentation compared to unprocessed mountain‐cultivated ginseng sprouts (MCGS). Among these, the concentration of ginsenoside CK, an essential bioactive substance in ginseng, is known to increase the most in FAMCGS (Lee et al. 2021, 2023; Ryu et al. 2022). FAMCGS also demonstrated enhanced antioxidant and anti‐inflammatory properties, with higher scavenging activities against radicals like ABTS and DPPH and more significant inhibitory effects on inflammatory mediators, including NO, MCP‐1, IL‐6, and TNF‐α (Lee et al. 2021). These improvements suggest that FAMCGS could be a potent natural source for nutraceutical applications and functional foods due to its enriched phytochemical profile and biological activities.

Ginsenoside CK is a secondary ginsenoside found in high concentrations in FAMCGS extract (FAMCGSE), resulting from fermentation and gut microbe interactions (Sharma and Lee 2020). CK has enhanced bioavailability and solubility compared to its precursor ginsenosides (Sharma and Lee 2020). It possesses a range of biological effects, including anti‐inflammatory, anti‐allergic, anti‐diabetic, neuroprotective, antiangiogenic, anti‐aging, anti‐cancer, and hepatoprotective properties in various in vitro and in vivo models (Liu, Zhu, and Wang 2022). However, high concentrations of CK in dietary supplements can be hepatotoxic (Liu, Zhu, and Wang 2022). Consuming CK through food sources or as part of a complex is advisable to mitigate these adverse effects. The mechanisms behind the anti‐inflammatory actions of CK‐rich FAMCGSE remain poorly understood (Lee et al. 2021, 2023). This study aims to explore the anti‐inflammatory effects of FAMCGSE in LPS‐stimulated RAW 264.7 macrophages and elucidate the underlying mechanisms.

## 2 Materials and Methods

### 2.1 Preparation of FAMCGSE and Determination of CK as Its Main Component

The fermented and aged mountain‐cultivated ginseng sprout extract (FAMCGSE) used in this study was the same as that analyzed in our previous study (Lee et al. 2023), and the concentrations were directly adopted from our earlier findings. The initial material, a five‐year‐old mountain‐cultivated ginseng sprout (MCGS), was obtained from Agricultural Corporation Ginseng‐Bio (Hamyang, Republic of Korea). The FAMCGSE was prepared using MCGS following established protocols detailed in the referenced study (Lee et al. 2023). The protocols included washing, steaming at 100°C for 60 min, aging at 75°C for 72 h (repeated three times), autoclaving at 121°C for 30 min, cooling to 30°C, and fermenting at 30°C for 5 days using bacterial strains *Lactoplantibacillus plantarum* P1201 and *Levilactobacillus brevis* BMK484. After fermentation, the sprouts were dried at 55°C for 3 days, ground into powder, and stored at −40°C. For ethanol extraction, 20 g of FAMCGS powder was extracted with 400 mL of 50% ethanol at 40°C ± 2°C for 5 h, filtered, concentrated to 20°Brix using a rotary evaporator, and filtered again.

High‐performance liquid chromatography chromatograms revealed 21 ginsenoside peaks in MCGSE and 16 peaks in FAMCGSE (Lee et al. 2023). In MCGSE, the ginsenosides were identified in ascending order as Re, Rb1, Rd., F2, Rd2, Ro, Rc, Rg1, Rb2, F3, Rg2, Rf, F1, F5, Rh1, PPD, CK, PPT, Rg3, Rb3, and Rh2. In FAMCGSE, the major ginsenosides were CK, F2, Ro, Rd2, Rg3, Rd., Rg2, and Rb1. Compared to MCGSE, FAMCGSE showed significant decreases in Rb1, Rc, Rb2, and Rd., and notable increases in F2, Rg3, and CK in FAMCGS. The significant ginsenosides in FAMCGSE were CK (2.22 mg/g), F2 (1.9 mg/g), and Rg3 (0.93 mg/g), with these concentrations showing 1.6‐fold, 6.64‐fold, and 11.7‐fold increases, respectively, compared to MCGSE. CK exhibited the most substantial increase, making it the predominant component of FAMCGSE.

### 2.2 Chemical Reagents

Unless specified, all chemicals were sourced from Sigma‐Aldrich (St Louis, MO, USA). Stock solutions of LPS (5 mg/mL) and dexamethasone (Dex; 10 mM) were prepared in distilled water. Compound K (CK; 100 mM), PD98059 (10 mM), SB203580 (25 mM), SP600125 (20 mM), BAY11‐7085 (50 mM), SR11302 (50 mM), and SP100030 (50 mM) were dissolved in dimethyl sulfoxide (DMSO). All solutions were diluted in the culture medium to achieve the desired working concentrations. The exact concentration of solvent was also added to the control group. The working concentrations of the chemicals were 30 μM for CK and 10 μM for PD98059, SB203580, and SP600125, 5 μM for BAY11‐7085, and 1 μM for SR11302 and SP100030. The 30 μM CK concentration was determined based on a previous study demonstrating that this concentration exhibits anti‐inflammatory effects without cytotoxicity (Ryu et al. 2018). The chemical concentrations used in this study were selected based on earlier studies that showed the effectiveness of these inhibitors at those levels (Ryu et al. 2015; Cho et al. 2019; Sun et al. 2017; Gerlag et al. 2000; Colombo et al. 2018).

### 2.3 Cell Culture and Viability Assay

The RAW264.7 macrophage cell line culture and cell viability assays were conducted following the procedures described in our previous study (Nyiramana et al. 2020). RAW 264.7 cell viability was assessed using the 3‐(4,5‐dimethylthiazol‐2‐yl)‐2,5‐diphenyltetrazolium bromide (MTT) reagent (5 mg/mL in phosphate‐buffered saline (PBS), Duchepa Biochem, Haarlem, The Netherlands). Cells were seeded at a density of 10^4^ cells/well (100 μL) in 96‐well plates and incubated for 24 h before treatment. Following this, cells were treated with chemicals, stimulated with LPS for 16 h, and/or pretreated with FAMCGSE for 2 h. Afterward, 10 μL of MTT solution (5 mg/mL) was added to each well and incubated in the dark at 37°C for 2 h. The supernatant was removed, and formazan crystals were dissolved in 100 μL of DMSO with 15 min of shaking at room temperature. Absorbance at 570 nm was measured using a VERSAmax microplate reader (Molecular Devices, San Pablo, CA, USA).

### 2.4 Nuclear Fast Red Staining and Spreading and Vacuolization Assay

The nuclear fast red staining solution was prepared by dissolving 0.1 g of nuclear fast red in 100 mL of 5% aluminum sulfate solution. The cells treated with chemicals were washed with phosphate‐buffered saline (PBS) and fixed at room temperature for 10 min. After fixation, the cells were washed with PBS three times and then stained with 0.1% nuclear fast red at room temperature for 3 min. The cells were observed under a microscope following a wash with distilled water. Images were captured using an Axiovert 40C microscope (Zeiss, Jena, Germany) to analyze cell spreading and vacuolization. A flattened and pseudopodia‐rich morphology characterized cell spreading, while vacuoles identified vacuolization within cells. The percentage of cells exhibiting each phenotype was calculated by dividing the number of affected cells by the total number of cells in a field of view. This process was performed separately for both spreading and vacuolization.

### 2.5 Measurement of Radical Scavenging and Enzyme Inhibition Activities

The radical scavenging activities of FAMCGSE against 2,2′‐azino‐bis(3‐ethylbenzothiazoline‐6‐sulfonate) (ABTS; Thermo Fisher Scientific, Ward Hill, MA, USA) and 2.2‐diphenyl‐1‐picrylhydrazyl (DPPH; Thermo Fisher Scientific) were evaluated using methods adapted from a previous study (Lee et al. 2021). The inhibitory effects of CK, F2, and Rg3 on cyclooxygenase‐2 (COX‐2) were assessed through modified methods from a previous study (Siregar et al. 2021).

### 2.6 Measurement of Intracellular Reactive Oxygen Species (ROS) Levels

Intracellular ROS levels were measured using two methods. RAW264.7 macrophages were seeded in poly‐L‐lysine‐coated image dishes (8 × 10^4^ cells/200 μL) and cultured for 24 h. Cells were then pretreated with FAMCGSE (0.01% and 0.1%) for 2 h, followed by stimulation with LPS (1 μg/mL) for 16 h. Intracellular ROS generation was measured using the 2′,7′‐dichlorodihydrofluorescein diacetate (H_2_DCFDA; Calbiochem, San Diego, CA, USA), which was added to the cells at a final concentration of 5 μM and incubated at 37°C for 30 min. After washing three times with PBS, cell fluorescence was visualized under a fluorescence microscope (Olympus, Tokyo, Japan). Cells cultured in black‐bottomed 96‐well plates were treated in the same manner, and ROS levels were quantified using a GloMax explorer multimode microplate reader (Promega, Medison, WI, USA).

### 2.7 Measurement of Nitrite Levels

Nitrite levels were determined using the Griess reagent system (Promega), as described previously (Jeong et al. 2016). RAW264.7 cells were seeded in 6‐well plates (7 × 10^5^ cells/well) and incubated at 37°C with 5% CO_2_ for 24 h. Cells were then pretreated with FAMCGSE for 2 h and stimulated with 1 μg/mL LPS for 16 h. The culture medium was collected and centrifuged at 2350 × *g* for 5 min. The supernatant was transferred to a 96‐well plate. Equal volumes of sulfanilamide solution were added to the samples and incubated for 8 min in dark conditions. Subsequently, 50 μL of N‐1‐naphthyl ethylenediamine dihydrochloride solution was added to each well and incubated at room temperature for 8 min. Absorbance was measured at 550 nm using a VERSAmax microplate reader (Molecular Devices). Nitrite levels were calculated from a standard curve generated using sodium nitrite.

### 2.8 Reverse Transcriptase‐Polymerase Chain Reaction (RT‐PCR) and Western Blotting Assays

Total RNA and protein isolation, as well as RT‐PCR and Western blotting assays, were conducted following the methods described in our previous study (Nyiramana et al. 2020). The PCR primer pairs used are listed in Table 1, with glyceraldehyde‐3‐phosphate dehydrogenase (GAPDH) serving as the loading control.

**TABLE 1:** Primer sequences used for PCR.

Gene name	GenBank acc. no.	Primer sequences (5′‐3′)		Expected size (bp)	Species
COX‐2	NM_011198.4	Sense Antisense	CCC AGA GCT CCT TTT CAA CCA TGC AGC CAT TTC CTT CTC TCC	381	Mouse
GAPDH	NM_017008.4	Sense Antisense	CTA AAG GGC ATC CTG GGC TTA CTC CTT GGA GGC CAT	201	Mouse
IL‐1β	NM_008361.4	Sense Antisense	GTT GAC GGA CCC CAA AAG AT TCG TTG CTT GGT TCT CCT TG	440	Mouse
IL‐6	NM_031168	Sense Antisense	CTT CAC AAG TCC GGA GAG GAG TGG TCT TGG TCC TTA GCC ACT	489	Mouse
iNOS	NM_010927	Sense Antisense	TTG CCC CTG GAA GTT TCT CTT TTG TCT CTG GGT CCT CTG GT	371	Mouse
TNF‐α	NM_013693.3	Sense Antisense	CAG CCT CTT CTC ATT CCT GC TGT CCC TTG AAG AGA ACC TG	339	Mouse

For Western blot analysis involving nuclear and cytoplasmic fractions, RAW 264.7 cells were subjected to the following protocol: Cells were homogenized in hypotonic lysis buffer and incubated on ice for 15 min. The cell lysates were then centrifuged at 142 × *g* for 5 min at 4°C to separate the nuclear and cytoplasmic fractions. The nuclear fraction was isolated by resuspending the pellets in a nuclear isolation buffer, adding 2% Triton X‐100, and centrifuging at 12,448 × *g* for 15 min at 4°C. The cytoplasmic fractions were obtained by centrifuging the initial supernatant at 15,401 × *g* for 20 min at 4°C and transferring the clear supernatant into a new 1.5 mL tube.

For Western blotting, the following antibodies were used: iNOS (#2982S, 1:1000 dilution, Cell Signaling, Danvers, MA, USA), COX‐2 (#4842S, 1:1000 dilution, Cell Signaling), NF‐κB p65 (#8242S, 1:1000 dilution, Cell Signaling), Lamin A (#ab8980, 1:1000 dilution, Abcam, Cambridge, UK), Phospho‐p44/42 MAPK (Erk 1/2, #4370S, 1:1000 dilution, Cell Signaling), p44/42 MAPK (#4695, 1:1000 dilution, Cell Signaling), Phospho‐p38 MAPK (#MA5‐15182, 1:1000 dilution, Thermo Scientific, Ward Hill, MA, USA), p38 MAPK (#2339S, 1:1000 dilution, Cell Signaling), Phospho‐JNK (#9251, 1:1000 dilution, Cell Signaling), JNK (#9252, 1:1000 dilution, Cell Signaling), α‐tubulin (#A11126, 1:1000 dilution, Thermo Scientific), and anti‐β‐actin (A5441, 1:5000 dilution, Sigma‐Aldrich). After overnight incubation with the primary antibodies at 4°C, secondary anti‐rabbit or anti‐mouse antibodies were used at a 1:5000 dilution (Assay Designs, Ann Arbor, MI, USA). Immunopositive bands were visualized using an enhanced chemiluminescence system (Thermo Fisher Scientific) and captured with an iBright CL1500 imaging system (Thermo Fisher Scientific/Life Technologies Holdings Pte Ltd., Singapore). Relative protein levels were quantified using β‐actin, Lamin A, or α‐tubulin as loading controls.

### 2.9 Enzyme‐Linked Immunosorbent Assay (ELISA) for Measurement of Proinflammatory Cytokines, Prostaglandin E2 (PGE_2_ ), and NF‐κB

The concentrations of IL‐1β, IL‐6, TNF‐α, and PGE_2_, as well as NF‐κB activity, were measured using ELISA kits according to the manufacturer's instructions and the methodology described in our previous studies (Siregar et al. 2021; Jeong et al. 2016). The proinflammatory cytokines (IL‐1β, IL‐6, and TNF‐α) and PGE_2_ in RAW264.7 cells were quantified using ELISA kits from R&D systems (Minneapolis, MN, USA). NF‐κB activation was assessed by measuring both phosphorylated (at serine 536) and total NF‐κB p65 protein using the NF‐κB p65 (pS536 + Total) ELISA Kit (#ab176663, Abcam). The absorbance of all plates was measured at 450 nm using a microplate reader (Molecular Devices).

### 2.10 Data Analysis and Statistics

The band intensities on agarose gels and Western blots were quantified using ImageJ software (version 1.51; National Institutes of Health, Bethesda, MD, USA). Data are presented as mean ± standard deviation (SD). Statistical analysis was performed using OriginPro2020 (OriginLab Corp., Northampton, MA, USA). Data normality was assessed, and subsequently, either one‐way ANOVA followed by the Bonferroni post hoc test (for normally distributed data) or the Kruskal–Wallis test followed by the Mann–Whitney test (for non‐normally distributed data) was applied to determine differences among groups. Statistical significance was set at *p* < 0.05.

## 3 Results

### 3.1 Inhibition of LPS‐Induced Activation in RAW 264.7 Cells by FAMCGSE

**FIGURE 1:**
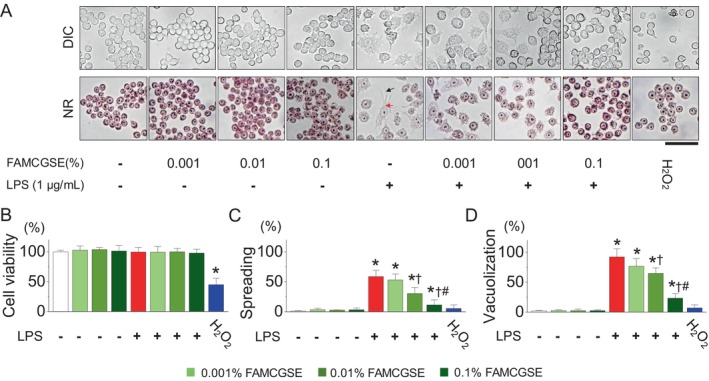
Effects of fermented and aged mountain‐cultivated ginseng sprout extract (FAMCGSE) on RAW 264.7 macrophage cell activation and viability in response to LPS treatment. (A) Morphological changes of RAW 264.7 cells exposed to LPS and treated with varying concentrations of FAMCGSE (0.001%–0.1%). The upper panel shows differential interference contrast (DIC) images, and the lower panel shows nuclear red (NR)‐stained images. The black and red arrows indicate LPS‐induced cell spreading and vacuoles in activated cells, respectively. Symbols are applied only to the LPS treatment group. Scale bars, 50 μm. (B) Effect of FAMCGSE on cell viability in response to LPS. (C, D) Analysis of the proportion of spread and vacuolized cells in response to LPS in the presence of FAMCGSE. Cells were pre‐treated with FAMCGSE (0.001% to 0.1%) for 2 h, followed by stimulation with 1 μg/mL of LPS for 16 h. Hydrogen peroxide (H_2_O_2_, 300 μM) treatment was used to control cell death induction. Bar graphs are presented as mean ± SD (*n* = 9). Plus and minus symbols represent with and without treatment, respectively. *p < 0.05 compared to control without LPS treatment. ^†^ *p* < 0.05 compared to LPS. ^#^ *p* < 0.05 compared to LPS + 0.01% FAMCGSE.

The effect of FAMCGSE on cell viability and morphological changes in RAW264.7 cells exposed to LPS was investigated. To assess the effect of FAMCGSE alone, RAW 264.7 cells were treated with various concentrations (0.001%–0.1%) of FAMCGSE for 24 h. To evaluate its protective effect against LPS‐induced activation, cells were pretreated with FAMCGSE for 2 h before stimulation with 1 μg/mL of LPS for 16 h. Differential interference contrast (DIC) and nuclear red (NR) staining images revealed that LPS treatment activated RAW264.7 cells, leading to cell spreading (black arrow) and vacuole formation (red arrow) (Figure 1A). FAMCGSE at these concentrations did not induce cell death under our culture conditions (Figure 1B). No significant morphological changes were observed in the FAMCGSE‐treated groups compared to the control (Figure 1A,C,D). The LPS‐only group exhibited large, flat cells with spreading and vacuoles, without cell death. Pretreatment with FAMCGSE significantly mitigated these LPS‐induced morphological changes in a dose‐dependent manner. Particularly, 0.01% and 0.1% FAMCGSE significantly reduced cell spreading and vacuolization compared to the LPS treatment alone (Figure 1C,D, *p* < 0.05). Additionally, hydrogen peroxide (H_2_O_2_) induced significant cell death but did not cause noticeable morphological changes compared to the control (Figure 1, *p* < 0.05).

### 3.2 Anti‐Inflammatory and Antioxidant Effects of FAMCGSE

**FIGURE 2:**
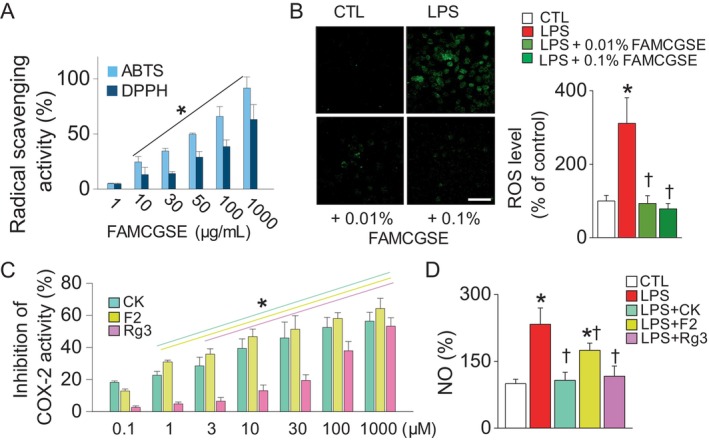
Anti‐inflammatory and antioxidant effects of FAMCGSE. (A) ABTS and DPPH radical scavenging activity of FAMCGSE at different doses. (B) Antioxidant effect of FAMCGSE on LPS‐induced ROS generation in RAW264.7 cells. The cells were pretreated with FAMCGSE (0.01% and 0.1%) for 2 h and stimulated with LPS (1 μg/mL) for 16 h. Intracellular ROS generation was determined using dichlorodihydrofluorescein (H_2_DCFDA), and the fluorescence intensity was measured using a microplate reader. Scale bars, 50 μm. (C) Inhibitory effect of main components of FAMCGSE on COX‐2 activity. CK and F2 at concentrations above 1 μM showed a significant difference from 0.1 μM, and Rg3 at concentrations above 3 μM showed significant difference from 0.1 μM. (D) Inhibitory effect of CK, F2, and Rg3 on LPS‐induced NO production. RAW264.7 cells were pretreated with FAMCGSE and then stimulated with 1 μg/mL LPS for 16 h. Culture media were collected to determine the amount of NO using the Griess reagent system. Bar graphs are presented as mean ± SD (*n* = 4). Plus symbols represent treatment with LPS. *p < 0.05 compared to control without LPS treatment. ^†^ *p* < 0.05 compared to LPS.

The antioxidant activity of FAMCGSE was assessed by measuring its ROS scavenging capability, while the anti‐inflammatory effects of its main components (CK, F2, and Rg3) were evaluated by determining their inhibition of COX‐2 activity and nitric oxide (NO) levels. FAMCGSE showed significant dose‐dependent scavenging activity against ABTS and DPPH radicals compared to 1 μg/mL FAMCGSE (Figure 2A, *p* < 0.05). Additionally, FAMCGSE reduced LPS‐induced ROS generation in RAW 264.7 cells, with significantly lower ROS intensity (DCF) observed in the 0.01% and 0.1% FAMCGSE pretreatment groups compared to the LPS‐only group (Figure 2B, *p* < 0.05). CK, F2, and Rg3 significantly inhibited COX‐2 activity in a dose‐dependent manner (Figure 2C, *p* < 0.05). Furthermore, pretreatment with CK, F2, and Rg3 at a concentration of 30 μM significantly reduced NO levels in LPS‐treated cells (Figure 2D, *p* < 0.05).

### 3.3 Reduction of Proinflammatory Mediators by FAMCGSE in LPS‐Stimulated Cells

**FIGURE 3:**
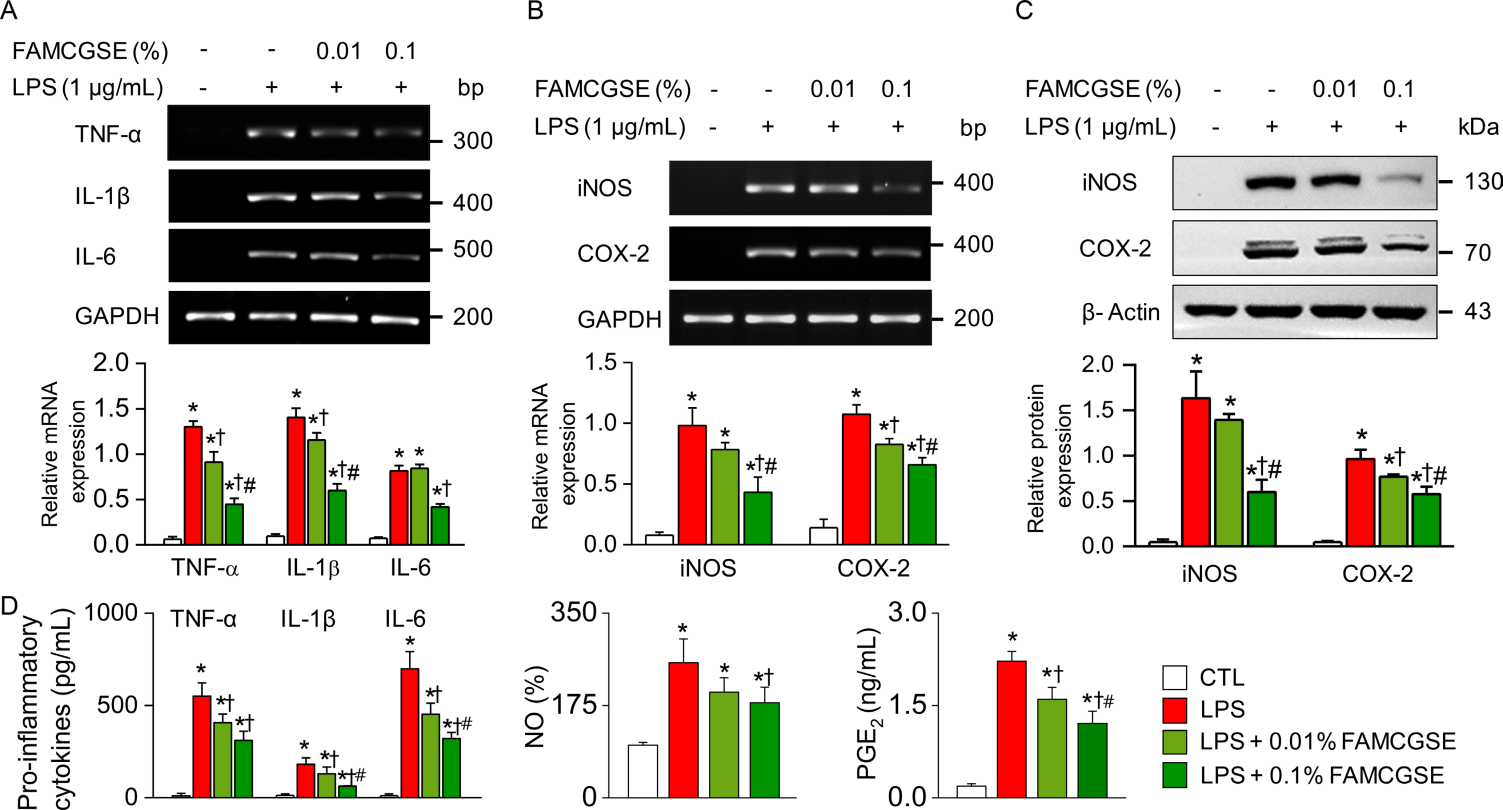
Suppression of proinflammatory mediators in LPS‐stimulated RAW 264.7 macrophages by FAMCGSE treatment. (A, B) Semi‐quantitative PCR analysis of TNF‐α, IL‐1β, IL‐6, iNOS, and COX‐2 mRNA expression levels. (C) Western blot analysis of iNOS and COX‐2 protein levels. The cells were pretreated with FAMCGSE at concentrations of 0.01% and 0.1% before LPS stimulation. GAPDH and β‐Actin were used as the loading control for PCR and Western blotting assays, respectively. (D) Concentration of TNF‐α, IL‐1β, IL‐6, NO, and PGE_2_ produced in LPS‐exposed cells. Bar graphs are presented as mean ± SD (*n* = 4). Plus and minus symbols represent with and without treatment, respectively. *p < 0.05 compared to control without LPS treatment. ^†^ *p* < 0.05 compared to LPS. ^#^ *p* < 0.05 compared to LPS + 0.01% FAMCGSE.

To assess the anti‐inflammatory effects of FAMCGSE, RAW 264.7 cells were pretreated with FAMCGSE for 2 h before a 16‐h exposure to LPS. Semi‐quantitative PCR analysis revealed that LPS treatment significantly increased the expression of TNF‐α, IL‐1β, IL‐6, iNOS, and COX‐2 compared to the control group (Figure 3A,B, *p* < 0.05). Pretreatment with FAMCGSE significantly reduced the mRNA levels of these inflammatory mediators. Western blot analysis confirmed that the elevated protein levels of iNOS and COX‐2 due to LPS were substantially decreased following pretreatment with 0.1% FAMCGSE (Figure 3C). These findings indicate that FAMCGSE effectively inhibits the expression of key proinflammatory mediators at both the transcriptional and translational levels. Furthermore, functional assays showed a significant decrease in the secretion of TNF‐α, IL‐1β, and IL‐6, as well as in NO production prostaglandin E_2_ (PGE_2_) levels in LPS‐stimulated RAW 264.7 cells (Figure 3D, *p* < 0.05), underscoring the potent anti‐inflammatory activity of FAMCGSE.

### 3.4 Inhibition of MAPK and NF‐κB Activation in Response to LPS by FAMCGSE and CK

**FIGURE 4:**
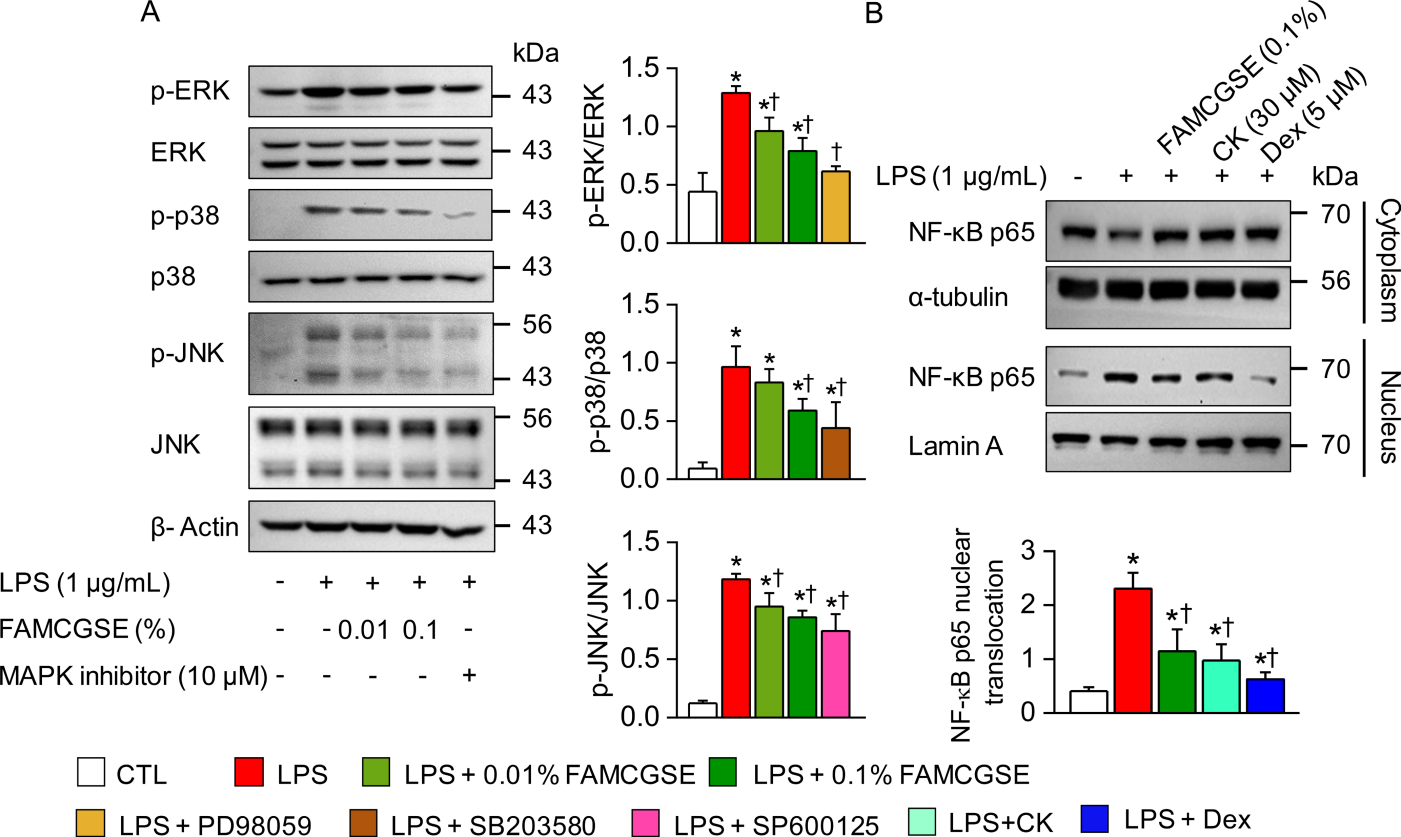
Effects of FAMCGSE on MAPK and NF‐κB p65 activation in LPS‐stimulated RAW 264.7 macrophages. (A) Western blot analysis showing the phosphorylation levels of MAPK proteins (ERK1/2, p38, and JNK) in LPS‐stimulated RAW 264.7 macrophages after treatment with FAMCGSE (0.01% and 0.1%). (B) Analysis of the nuclear and cytoplasmic distribution of NF‐κB p65 in LPS‐stimulated RAW 264.7 macrophages treated with FAMCGSE or CK. Lamin A and α‐tubulin were used as nuclear and cytoplasmic markers, respectively. The accompanying bar graph indicates that FAMCGSE significantly decreased the nuclear translocation of NF‐κB p65. Data are presented as mean ± SD of four independent experiments. *p < 0.05 compared to control without LPS treatment. ^†^ *p* < 0.05 compared to LPS.

The effects of FAMCGSE on the regulation of mitogen‐activated protein kinase (MAPK) activation, which is crucial for cellular stress responses, were evaluated. The analysis showed that LPS stimulation significantly activated extracellular signal‐regulated kinase 1/2 (ERK), p38, and c‐Jun N‐terminal kinases (JNK) (*p* < 0.05). Pretreatment with 0.01% FAMCGSE decreased the phosphorylation of ERK and JNK, while 0.1% FAMCGSE reduced the activation of all three MAPKs induced by LPS (Figure 4A). MAPK inhibitors PD98059 (ERK), SB203580 (p38), and SP600125 (JNK), each at 10 μM, effectively reduced LPS‐induced MAPK activation.

In addition, the translocation of NF‐κB into the nucleus, an essential step in the inflammatory response, was examined. After LPS activation, NF‐κB, sequestered in the cytoplasm, is translocated into the nucleus. Analysis of nuclear and cytoplasmic fractions showed that pretreatment with FAMCGSE (0.1%) and CK (30 μM) significantly inhibited LPS‐induced nuclear translocation of NF‐κB p65 (Figure 4B, *p* < 0.05). The inhibitory effects of CK and FAMCGSE were comparable to those of dexamethasone (5 μM), a known anti‐inflammatory agent.

**FIGURE 5:**
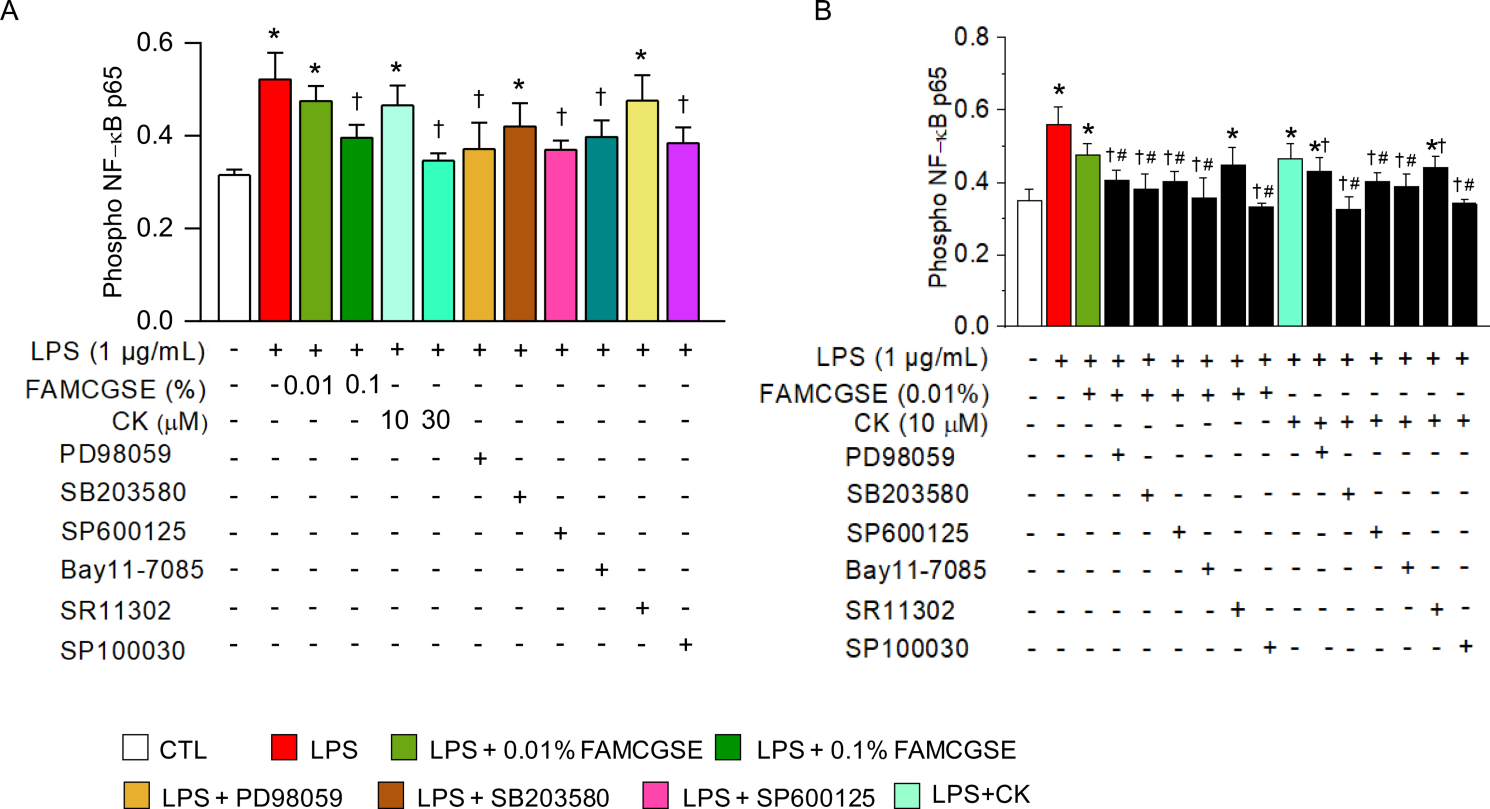
Reduction of NF‐κB activation by MAPK inhibition. (A) ELISA results showing the phosphorylation of NF‐κB p65 in RAW 264.7 cells treated with FAMCGSE, CK, or specific MAPK inhibitors (PD98059, SB203580, SP600125) or NF‐κB and AP‐1 inhibitors (Bay11‐7085, SR11302, SP100030). (B) Combined effects of FAMCGSE or CK and MAPK inhibitors on NF‐κB activation induced by LPS in RAW264.7 cells. Data are presented as mean ± SD of three independent experiments. Plus and minus symbols represent with and without treatment, respectively. *p < 0.05 compared to control without LPS treatment. ^†^ *p* < 0.05 compared to LPS. ^#^ *p* < 0.05 compared to LPS + 0.01% FAMCGSE or LPS + CK.

To explore the relationship between MAPK and NF‐κB in the anti‐inflammatory effects of FAMCGSE and CK, an NF‐κB phosphorylation ELISA assay was performed in the presence of MAPK inhibitors. Pretreatment with FAMCGSE (0.1%) and CK (30 μM) suppressed NF‐κB phosphorylation compared to the LPS group. As expected, NF‐κB inhibitors Bay11‐7085 (10 μM) and SP100030 (10 μM) inhibited NF‐κB phosphorylation. Similarly, ERK and JNK inhibitors reduced NF‐κB phosphorylation, while the p38 inhibitor and SR11302 (an AP‐1 inhibitor) did not significantly affect it (Figure 5A). The combination of FAMCGSE (0.01%) or CK (10 μM) with MAPK and NF‐κB inhibitors significantly enhanced the effects of FAMCGSE and CK, except when combined with SR11302 (Figure 5B, *p* < 0.05). This combination led to an increased suppression rate compared to treatment with inhibitors alone, demonstrating a synergistic effect at low doses of FAMCGSE or CK.

## 4 Discussion

Ginseng has long been prized for its medicinal properties, including anti‐inflammatory, antioxidant, and immune‐enhancing effects (Saba et al. 2018; Park et al. 2012; Kim et al. 2012; Lee et al. 2019). Numerous studies have uncovered the molecular mechanisms behind ginseng's therapeutic potential, raising its profile as a valuable health supplement. However, due to the lengthy cultivation period and high cost of ginseng, efforts have been made to develop alternatives that require less time to grow and provide similar nutritional benefits at a lower price. One such alternative is FAMCGSE. This study is the first to elucidate the molecular mechanisms underlying FAMCGSE's anti‐inflammatory properties. While previous studies have noted the antioxidant benefits of FAMCGSE, the mechanisms were not fully understood (Lee et al. 2021, 2023). By exploring these anti‐inflammatory mechanisms, this study aims to enhance the value of FAMCGSE as a potent alternative to traditional ginseng.

Due to the meager yield of domestic wild ginseng, it is challenging to process and fully utilize its benefits effectively. To overcome this, we cultivated ginseng under conditions resembling its natural wild ginseng habitat. By integrating fermentation and aging processes, we enhanced the production of CK, a ginsenoside present in higher concentrations in wild ginseng than in cultivated varieties, leading to the creation of FAMCGS. Consequently, FAMCGSE is emerging as a superior dietary supplement that harnesses the combined benefits of both wild and cultivated ginseng. The distinct ginsenoside profiles of wild and cultivated ginseng stem from differences in growing conditions and environmental stressors. Wild ginseng is often regarded as superior in medicinal quality, likely due to its higher concentrations and greater diversity of ginsenosides, including rare types such as Rk1, F1, Rg5, Rh1, CK, Rc, Rb1, Rd., Rf, Rg1, and others, such as notoginsenoside H, glucoginsenoside Rf, notoginsenoside R1, and pseudoginsenoside RT2, along with compounds like stigmasterol and β‐sitosterol (Ma et al. 2023; Liu et al. 2022; Jeong et al. 2010). Conversely, cultivated ginseng, typically grown under controlled conditions, tends to have higher levels of compounds like chicusetsusaponin IVa, malonylginsenoside Rd., pseudoginsenoside Rc1, malonylfloralginsenoside Rd6, malonylginsenoside Rb1, Rd., Rb2, and Re (Liu et al. 2022; Jeong et al. 2010).

Typically, macrophages release inflammatory mediators when the body encounters an infection to combat the invading pathogens (Fujiwara and Kobayashi 2005b). LPS from bacteria binds to TLR4, triggering downstream inflammatory signaling pathways that produce inflammatory mediators like iNOS and COX‐2. These mediators subsequently activate a cascade of proinflammatory cytokines, including TNF‐α, IL‐1β, and IL‐6 (Saba et al. 2018). In LPS‐stimulated RAW 264.7 cells, FAMCGSE significantly reduced the levels of iNOS and COX‐2, both at the transcriptional and translational levels, and decreased the production of these proinflammatory mediators. This reduction suggests that FAMCGSE could have powerful anti‐inflammatory properties.

The activation of NF‐κB by LPS initiates an inflammatory cascade involving cytokines, adhesion molecules, and various REL/NF‐κB and IκB members (Grilli, Chiu, and Lenardo 1993). In this study, treatment with FAMCGSE and CK mitigated LPS‐induced NF‐κB phosphorylation. Additionally, these treatments also influenced the MAPK pathways, which are activated by the stress of LPS‐TLR4 binding (Chen et al. 2018; Li et al. 2022; Lu et al. 2019). FAMCGSE reduced the phosphorylation of MAPKs (ERK1/2, p38, JNK) in RAW 264.7 cells (see Figure 5A). Similarly, CK has been shown in previous studies to reduce LPS‐induced MAPK activation (Ryu et al. 2018; Lu et al. 2019). Moreover, LPS‐induced NF‐κB activity was suppressed in cells treated with MAPK inhibitors. Interestingly, 0.1% FAMCGSE significantly diminished p38 activity, while 0.01% FAMCGSE had no effect, and the p38 inhibitor alone did not alter NF‐κB activity. However, co‐treatment with 0.01% FAMCGSE and the p38 inhibitor significantly decreased NF‐κB activity compared to either treatment alone. These results suggest that LPS‐induced activation of the MAPK pathway in RAW264.7 cells plays a crucial role as an upstream signal for NF‐κB activation (Chen et al. 2022; Schulze‐Osthoff et al. 1997; Wang et al. 2014), and FAMCGSE appears to inhibit NF‐κB phosphorylation by acting both as a MAPK inhibitor and as an enhancer of MAPK inhibitor effects.

The anti‐inflammatory effects of CK, the main component of FAMCGSE, are well established (Ryu et al. 2022; Liu, Zhu, and Wang 2022; Chen et al. 2019; Kim et al. 2017), making the inferred anti‐inflammatory benefits of FAMCGSE plausible. In this study, both FAMCGSE and CK inhibited NF‐κB p65 activation, with no significant difference observed between the two groups. However, FAMCGSE may be more effective when considering CK concentration alone. The CK concentration in 0.1% FAMCGSE (3.5 μM CK) is approximately eight times lower than that of CK alone (30 μM) used in the experiments. This enhanced efficacy may be due to the synergistic interaction of other ginsenosides in FAMCGSE, such as F2 and Rg3, both of which decreased COX‐2 activity in a concentration‐dependent manner and significantly reduced LPS‐induced NO production. Additionally, the anti‐inflammatory effects of FAMCGSE may be further amplified by its antioxidant properties, which have been shown to reduce ROS in cells. These combined properties of FAMCGSE provide a strong foundation for future clinical studies exploring its potential as a natural therapeutic agent. Its accessibility and ability to deliver comparable benefits to conventional ginseng at a lower cost position FAMCGSE as a valuable alternative in anti‐inflammatory treatment strategies.

In conclusion, our findings demonstrate that FAMCGSE exhibits potent anti‐inflammatory effects in LPS‐stimulated RAW 264.7 macrophages. FAMCGSE effectively suppressed proinflammatory mediators and inhibited the MAPK and NF‐κB pathways, highlighting its potential as a promising anti‐inflammatory agent.

## Author Contributions

**Dang Long Cao:** investigation (lead), visualization (equal), writing – original draft (supporting). **Min‐Seok Woo:** methodology (supporting). **Eun‐Jin Kim:** methodology (equal). **Byeonggyu Ahn:** investigation (supporting). **Anjas Happy Prayoga:** investigation (supporting). **Sang Soo Kang:** funding acquisition (supporting), writing – review and editing (supporting). **Kye Man Cho:** funding acquisition (equal), resources (lead). **Dawon Kang:** conceptualization (equal), data curation (equal), formal analysis (equal), funding acquisition (equal), methodology (equal), supervision (lead), visualization (equal), writing – review and editing (lead).

## Conflicts of Interest

The authors declare no conflicts of interest. The funding sponsors did not influence the study design, data collection, analysis, interpretation, manuscript writing, or publication of the results.

## Data Availability

The data supporting the findings of this study are available upon reasonable request to the corresponding author (D.K.). All pertinent data underpinning the conclusions of this research are included within the article.
